# Allosteric effects of the coupling cation in melibiose transporter MelB

**DOI:** 10.1101/2025.07.10.664195

**Published:** 2025-07-15

**Authors:** Parameswaran Hariharan, Yuqi Shi, Rosa Viner, Lan Guan

**Affiliations:** 1 Department of Cell Physiology and Molecular Biophysics, Center for Membrane Protein Research, School of Medicine, Texas Tech University Health Sciences Center, Lubbock, TX, USA; 2 Thermo Fisher Scientific, San Jose, CA, USA

**Keywords:** Allostery, symport, structural dynamics, sugar recognition, x-ray crystallography, HDX-MS

## Abstract

The major facilitator superfamily (MFS) transporters play significant roles in human health and disease. *Salmonella enterica* serovar Typhimurium melibiose permease (MelB_St_), which catalyzes the symport of galactosides with Na^+^, H^+^, or Li^+^, is a prototype of this important transporter superfamilies. We have published the structures of the inward- and outward-facing conformations of MelB_St_ with galactoside or Na^+^ bound, and determined the binding thermodynamic cycle. We have proposed that positive cooperativity between the two co-transported solutes plays a key role in the symport mechanism of MelB_St_; however, the molecular basis for this core mechanism remains unclear. In this study, we investigated the structural dynamics induced by melibiose, Na^+^, or both on MelB_St_ using hydrogen-deuterium exchange mass spectrometry (HDX-MS). We also refined the specific determinants for the sugar recognition in both protein and galactoside molecules by solving the crystal structures of D59C MelB_St_ bound to melibiose and two other sugars that contain different numbers of sugar units, and identified a critical water molecule as part of the specific determinants from a α-NPG-bound structure. Our integrated structural and HDX-MS analyses support the notion that the binding of the coupling cation at a remote site stabilizes those dynamic sidechains in the sugar-binding pocket, leading to a high-affinity state. This study provides the molecular basis for the essential symport mechanism through positive cooperativity, which may serve as a general mechanism for most cation-coupled symporters.

## Introduction

The Solute Carrier (SLC) family of transporters encompasses diverse superfamilies of membrane proteins with various protein folds and employing different transport mechanisms to facilitate the translocation of a wide range of solutes across cell membranes (1). The largest superfamily of SLC transporters is the major facilitator superfamily (MFS)(2). MFS transporters comprise a significant number of cation-coupled secondary active transporters and are responsible for the uptake of a broad spectrum of solutes across cell membranes, playing crucial roles in physiology, pathology, and pharmacokinetics, and are emerging as drug targets (3, 4). Recent rapid advancements in membrane protein research have greatly enhanced our understanding of protein conformation and mechanisms(5–8); however, critical details remain lacking. For example, in cation-coupled symport, it is still unclear how the coupling cations help in the binding, translocating, and accumulating the primary substrate.

For MFS secondary active transporters, most members use H^+^ as the coupling cation, and a few members use Na^+^, such as the Na^+^-coupled lipid transporter (MFSD2A)(9, 10) that is expressed in the major organ barriers, including the blood-brain barrier or blood-retina barrier. MFSD2A plays a critical role in the uptake of essential lipids into these neural tissues (9–11). The Na^+^-coupled melibiose transporter of *Salmonella enterica* serovar Typhimurium (MelB_St_), which is a well-characterized representative for the Na^+^-coupled MFS transporters, catalyzes the symport of a galactopyranoside with Na^+^, H^+^, or Li^+^, and is a useful model system for studying cation-coupled transport mechanisms (12–22). Two major conformations of MelB_St_ have been determined: one is an outward-facing conformation at apo or galactoside-bound states(20, 22), and the other is an inward-facing conformation at a Na^+^-bound state(21). The primary substrate-specificity determinant pocket and the cation-specificity determinant pocket have been structurally and functionally characterized (19–23). Binding at both pockets exhibits a positive cooperativity; that is, the binding of one increases the affinity for the other (19). All three coupling cations compete for the same binding pocket, and the transport stoichiometry is 1 galactoside: 1 cation (Na^+^, H^+^, or Li^+^). The sugar affinity depends on the cation identity, with the cooperativity numbers (fold of increase in affinity) being 8, 5, or 2 for Na^+^, Li^+^, and H^+^, respectively (19, 20). Notably, the sugar-binding affinity is also dependent on MelB_St_ conformation (21), in addition to being sensitive to the binding of the coupling cation and its identity. By trapping MelB_St_ in an inward-facing state using the inward-facing conformation-specific binder nanobody-725 (Nb725), both experimental sugar-binding assays and cryoEM structural analysis support a low-affinity state of the sugar-binding pocket (21, 23). Remarkably, Na^+^ binding to the inward-facing conformation remains unchanged (21, 23). These results provide experimental evidence supporting the previously proposed stepped-binding kinetic model for melibiose/Na^+^ symport, in which Na^+^ binds first and is released after the sugar release on the opposite surface (15, 18, 24, 25).

Positive cooperativity is proposed to be the key symport mechanism in MelB_St_, but the molecular basis for this critical mechanism remains unclear. The structures indicate that the bound sugar and Na^+^ have no direct contact, while the two binding pockets are in close proximity (20, 21). The minimum free-energy landscape for sugar translocation has been simulated based on the structures of the outward- and inward-facing conformations as two starting points (26). The results suggest that the Na^+^ contribution to the binding free energy of sugar by direct contact is negligible. The cooperativity between melibiose and Na^+^ occurs through allosteric coupling, primarily via electrostatic effects. To experimentally evaluate this concept, in this study, we enhanced the resolution of the structure with α-NPG bound, which revealed a critical water molecule, and also determined crystal structures with three other α-sugars (melibiose, raffinose, or α-MG) that contain different numbers of sugar units to verify the sugar-recognition specific determinants. We also analyzed the structural dynamics with HDX-MS induced by melibiose, Na^+^, or both on MelB_St_. The extensive integrated analyses of the structure and structural dynamics by MD simulations and HDX-MS support the conclusion that the binding of the coupling cation at a remote location stabilizes the sugar-binding residues to switch to a higher-affinity state. Therefore, the coupling cation in this symporter is an allosteric activator.

## Results

### Outward-facing structures of MelB complexed with varied sugar substrates

**Fig. 1.**
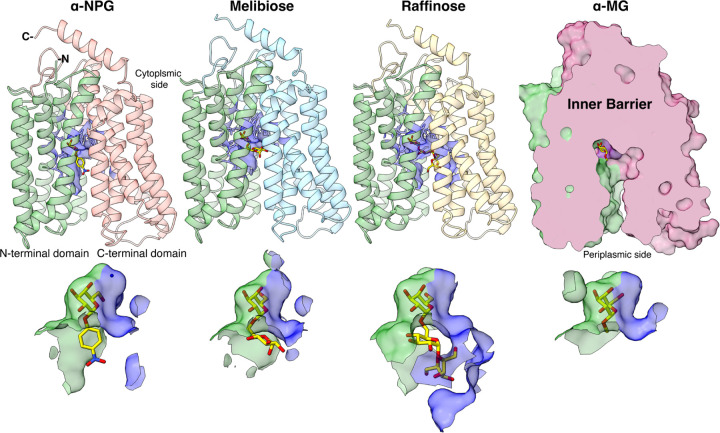
Crystal structure of D59C MelBSt in complex with α-sugars or sugar analogs. The identities of the bound sugars are indicated at the top. Upper row: Cartoon representation of the structures of D59C MelB_St_ bound with α-nitrophenyl galactoside (α-NPG), melibiose (α-disaccharide), and raffinose (α-trisaccharide), respectively, along with a surface presentation of D59C MelB_St_ complexed with α-methyl galactoside (α-MG). All structures were oriented with the cytoplasmic side on top and the N-terminal domain (colored green) on the left. Each sugar molecule is colored yellow. The blue sticks and surfaces indicate residues within 5 Å of the sugar molecules. Lower row: the sugar-binding pockets. Residues from the N-terminal and C-terminal domains were shown in surface representation, and colored in green and blue, respectively.

We have published the crystal structures of MelB_St_ with bound sugar analogues α-NPG or melibiose-derived detergent (DDMB), from a MelB_St_ uniport mutant D59C (20), or a solely H^+^-coupled symporter mutant D55C(22). Here we report four crystal structures of D59C MelB_St_ with the bound endogenous sugar substrate melibiose, and two other α-galactosides, α-methyl galactoside (α-MG) with a single sugar unit or raffinose with three sugar units, as well as α-NPG at an improved resolution. The statistics of the four structures are presented in Table S1. All outward-facing structures are virtually identical with RMSD values of less than 0.4 (Fig. S1). This typical MFS-fold transporter has 12 transmembrane helices and is organized into two six-helix domains linked by the middle loop between helices VI and VII (Loop_6–7_). There are two cytoplasmic helices on the middle loop or the C-terminal tail or lid (Fig. 1). In all structures, one sugar molecule is bound in the middle of the protein and sandwiched by the helices from both the N- and C-terminal domains. As indicated by the sliced surface, the binding residues located within the cytoplasmic leaflet of both domains shape the edge of the inner barrier that prevents the sugar from passing across the transporter into the cytoplasm. On the periplasmic side, there is an open vestibule connecting the solvent to the binding pocket.

#### α-NPG binding refined to a resolution of 2.7 Å.

**Fig. 2.**
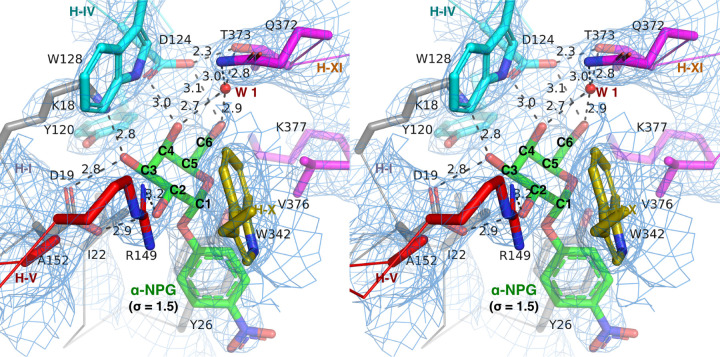
α-NPG binding. The cross-eye stereo view of α-NPG binding. **(a)** Isomesh map of the α-NPG-binding site, contoured at a level of σ =1.5. Dashed lines indicate distances within hydrogen-bonding or salt-bridge interactions (Å). W1, water molecule 1 in red. The helices are labeled with Roman numerals.

The improved resolution from 3.01 Å to 2.7 Å provides a better-resolved density map for the bound α-NPG molecule, which further supports the originally assigned pose (Fig. 2) (20). As described, the binding pocket is formed by 14 residues on five helices, including N-terminal helices I (Lys18, Asp19, Ile22, and Tyr26), IV (Tyr128, Asp124, Tyr120), and V (Arg149 and Ala152), and the C-terminal helices X (Trp342) and XI (Gln372, Thr373, Val376, and Lys377).

A water molecule was modeled to a positive density, which located at a good hydrogen-bonding distances from the C4-OH on the galactopyranosyl ring and Thr733 on helix XI, and also surrounded by C6-OH, Gln372 on helix XI and Asp124 on helix IV (Fig. 2). Overall, the galactosyl moiety is primarily stabilized by the N-terminal charged residues Asp19, Asp124, and Arg149. Especially, strong and continuous density between Asp19 and the C2-OH and C3-OH and Asp124 with C4-OH were observed in the previous maps (20) and current maps, indicating that both negatively charged Asp resides and the water molecule play critical roles in the binding.

Five more water molecules were modeled, and three interact with the previously identified charge network involved in 9 charged residues between the N- and C-terminal domains (Fig. S2a, b). The water 2 and 3 interact with the charged pair Arg295 (helix IX) and Glu351 (helix XI) and the water 4 interacts with Arg138 on helix IV, which also form a salt bridge with Glu142 to stabilize the hydrogen-bonding interaction between Glu142 and Arg295 (Fig. S2b).

#### Melibiose binding.

**Fig. 3.**
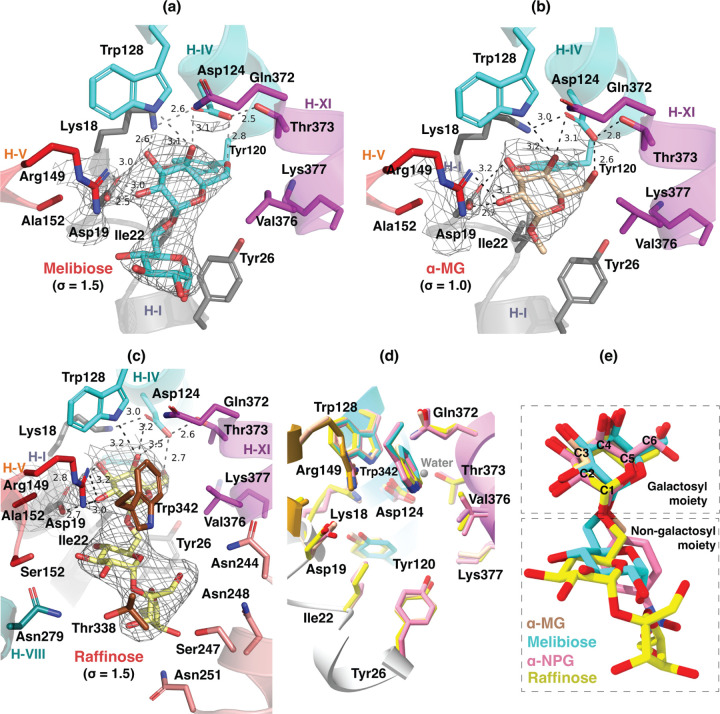
Binding of melibiose, α-MG, and raffinose. All residues within a 5-Å distance to the bound substrates were shown in sticks. Isomesh maps for each sugar and Asp19 were contoured at levels of σ = 1.5 for melibiose and raffinose or σ = 1.0 for α-MG. Dashed lines, the distances within hydrogen-bonding and salt-bridge interactions (Å). **(a)** Melibiose binding. **(b)** α-MG binding. **(c)** Raffinose binding. **(d)** Residues in the sugar-binding pockets from the alignment of all four structures. **(e)** Substrates from the alignment of all four structures. Carbon positions on the galactosyl (C1–6) and glucosyl moiety (C1–6′) are labeled on the melibiose molecule. Trp342 was removed for clarity in panels a, b, and d.

The endogenous substrate melibiose is formed from a galactose unit and a glucose unit linked by an α−1,6 galactosyl bond (d-Gal-(α1→6)-d-Glc). The melibiose-bound D59C MelB_St_ structure was refined to a resolution of 3.05 Å, and the density map displayed a clear two-unit blob in the binding pocket, fitting well with one molecule of melibiose. While the water molecule was not modeled due to limited resolution, a positive density is also present at W1 position. Two waters at W2 and W3 positions were also modeled in this structure. Interestingly, the binding affinity between melibiose and α-NPG differs by a factor of 100; the only difference in the binding sites is the pose and chemistry of the non-galactosyl moiety (Fig. 3a, e). In α-NPG, the hydrophobic phenyl group, positioned at 4 Å-distance from the phenyl group of Tyr26, forms a strong stacking interaction. While melibiose has four additional OH groups, there is no further polar interaction with MelB. This glucosyl ring rotates away and becomes nearly perpendicular to the phenyl ring of Tyr26 (Fig. S3). The absence of aromatic stacking may contribute to the relatively poor affinity of melibiose.

#### α-Methyl galactoside binding.

This sugar molecule has a methyl substituent at the anomeric C1 position in an α-linkage. The α-MG-bound structure of the D59C MelB_St_ mutant was refined to a resolution of 3.68 Å, and the density map still clearly displayed a one-unit blob in the binding pocket, where one α-MG was modeled similarly to the galactosyl moiety of melibiose (Fig. 3b).

#### Raffinose binding.

Raffinose is a trisaccharide formed from melibiose and fructose. The raffinose-bound D59C MelB_St_ mutant structure was refined to a resolution of 3.40 Å, and the density map displayed a clear three-unit blob in the binding pocket, fitting well with this trisaccharide. As expected, the raffinose-binding pocket is significantly larger than that of melibiose and α-NPG (Fig. 3c), involving two additional helices. Consequently, a set of polar side chains on helix VII (Asn244, Ser247, Asn248, and Asn251), helix VIII (Asn279), and helix V (Ser153) were found to be within 4–5 Å of either the glucosyl or fructosyl moieties. Interestingly, the polar interactions at hydrogen-bonding distances between raffinose and MelB_St_ are nearly identical to those in α-MG, melibiose, or α-NPG, with most occurring within the galactosyl moiety, whiles the Asp19 is at a hydrogen-bonding distance from the OH-4 on the glucosyl moiety.

All four galactosyl moieties align well in the specificity-determinant pocket, regardless of the number of monosaccharide units (Fig. 3d, e; Fig. S1). This alignment strongly supports the previous conclusion that the galactosyl moiety determines the specificity of the primary substrates; however, while the non-galactosyl moiety contributes to the binding affinity. The galactosyl-binding pocket is well formed with little room for flexibility in the sugar pose; however, the spacious cavity can accommodate at least one more fructose unit, as shown in the raffinose.

### Isothermal titration calorimetry (ITC) measurement of the binding affinity

**Fig. 4.**
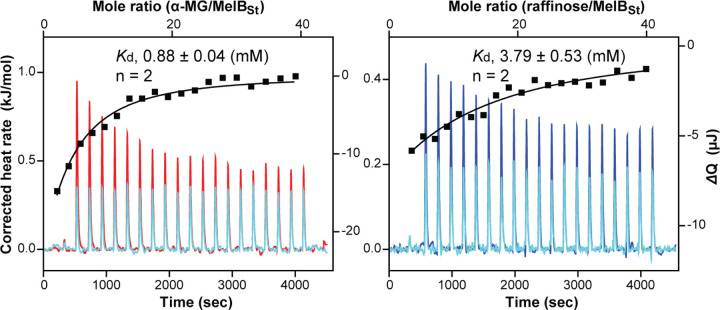
Binding affinity determination via ITC. The binding measurement of α-MG or raffinose to the WT MelB_St_ was performed with a NanoITC calorimeters (TA Instruments) at 25 °C as described in Methods. The thermograms were plotted as baseline-corrected heat rate (μJ/sec; left axis) vs. time (bottom axis) for the titrant to MelB_St_ (red for α-MG and blue for raffinose) or to buffer (light blue). The heat change ∆*Q* (μJ; filled black symbol) was plotted against the mole ratio of the sugar to WT MelB_St_ (top/right axes). **(a)** α-MG. (b) Raffinose.

Binding affinity *K*_d_ values of the WT and D59C MelB_St_ for melibiose of 1.25 mM ± 0.05 mM and 4.96 ± 0.11 mM, respectively, and for α-NPG of 16.46 ± 0.21 μM or 11.97 ± 0.09 μM, respectively, have been determined by ITC (19, 20, 25, 27). To determine the binding affinity of MelB_St_ for α-MG and raffinose, ITC measurements were also utilized to determine the *K*_d_ values in the presence of Na^+^ (Fig. 4). By injecting the raffinose or α-MG in a buffer-matched solution into the sample cell containing the WT MelB_St_ in the presence of Na^+^, the isotherm curve fitted well, yielding *K*_d_ values of 0.88 ± 0.04 mM or 3.79 ± 0.53 mM or for α-MG or raffinose, respectively. It is surprising that raffinose, which has more sugar units, exhibits a poor binding affinity to MelB_St_.

### Structural dynamics measured by HDX-MS

To determine the structural dynamics of MelB_St_ in response to the substrate and/or Na^+^, the bottom-up HDX-MS method was employed to measure the exchange rates of amide hydrogen with deuterium by analyzing time-dependent mass spectra at the peptide level as described(28–30). Three pairs of datasets were collected, including Apo vs. melibiose, Na^+^, or both, as outlined in the Methods section. The analyzed region of MelB_St_ spans positions 2–470 out of 476, achieving a coverage of over 87.47% and 86.62% from 149 or 152 overlapping deuterium-labeled peptides (Table S2; Fig. S4). There are 59, 63, or 59 non-covered residues for the datasets of Apo vs. melibiose-bound, Na^+^-bound, and melibiose- and Na^+^-bound states, respectively. Most of these positions are localized within the transmembrane regions, particularly on the C-terminal half (Table S4). Interestingly, the two longest helices, IV and X, display a significantly higher number of non-covered positions compared to the other helices. While a significant number of such positions are present on the cytoplasmic loops, all periplasmic loops are covered by labeled peptides.

The HDX rates are varying in different regions (Table S4). When averaging the related deuterium uptake (D%) from all peptides at all time points at a given region, the N-terminal helices I and V, as well as the C-terminal helices VIII and IX exhibited higher values, indicating they are more dynamic regions of Apo MelB_St_.

**Fig. 5.**
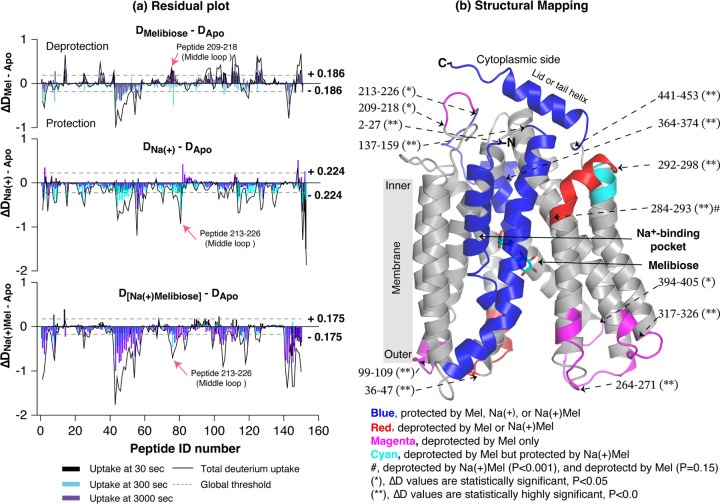
HDX-MS. HDX experiments on WT MelB_St_ in Apo or Holo states (with melibiose, Na^+^, or Na^+^ plus melibiose) were conducted as described in Methods. **(a)** Residual plots (D_Holo - Apo_). Differential deuterium uptakes ΔDs, calculated from paired conditions for position 2–470, were plotted against overlapping peptides for each time point and the total uptake. Black, cyan, and purple bars, the deuterium uptake at 30, 300, 3000 sec, respectively; dark gray curve, total uptake from all three time points. Deprotection, ΔD_Holo – Apo_ > 0; protection_,_ ΔD_Holo – Apo_ < 0. Each sample was analyzed in triplicates. Regions not covered by labeled peptides were excluded. Dashed lines, the global threshold values calculated from each dataset. **(b)** Mapping onto the 3D structure of the melibiose-bound state. Peptides with ΔD value greater than the absolute value of the threshold and P < 0.05 at any time point were colored according to the legend on the figure. The membrane region of MelB_St_ is indicated by a gray bar. Each peptide or overlapping peptide was indicated by arrows pointing to the start of the peptide. The helices I, V, VIII, and IX, as well as the C-terminal helix (lid), are labeled individually. Mel, melibiose.

The differential deuterium labeling (ΔD) was calculated based on the deuterium uptake in the absence (Apo state) or presence of specific ligand(s) (Holo state), and is presented as the residual plot of each peptide between the two states (_Holo – Apo_) (Fig. 5a) at three labeling time points and the sum of all. The dashed lines indicate the global thresholds (ΔD) for each dataset. The C-terminal His tag and tail residues at positions 470–475 are highly dynamic and fully labeled with greater variations and are excluded from the analysis. The residual plots include data from all peptides, and the ΔD values greater than the global threshold and P values <0.05 are considered statistically significant. Melibiose binding induced a wide range of structural effects on MelB_St_ throughout the entire MelB_St_ polypeptide, and the effects include protection (less deuterium uptake in the Holo state) and deprotection (greater deuterium uptake in the Holo state). In contrast, Na^+^ alone or in combination with melibiose primarily caused deprotection. As shown in Table S3, more than 50% of residues (237, 264, or 257 positions) exhibited either insignificant ΔD values (D_Mel - Apo_ < |0.186|, ΔD_(Na+)_ - Apo < |0.224|, or ΔD_Na(+)Mel_ - Apo < |0.175|) or greater P values (> 0.05). A peptide with at least one significant ΔD or overlapping peptides extending from the peptides with at least one significant ΔD were mapped onto the melibiose-bound structure of each dataset individually (Fig. S5). The uncovered positions were indicated in gray spheres, and the positions with insignificant ΔD values are shown in backbone representation. Peptides with significant ΔD from all three datasets are presented together in Figure 5b.

Consensus effects are observed from all three conditions. Interestingly, peptides with significant ΔD values are clustered in several regions. Nearly full-length transmembrane helices I and V, as well as the tail helix, demonstrated protections from melibiose, Na^+^, or Na^+^ with melibiose. In particular, the peptides containing sugar-binding critical residues (Lys18, Asp19, and Arg149 on helices I and V) showed statistically high confidence (P < 0.01, Fig. 5b, Blue). The peptide 213–226 in the middle loop across the three conditions was also protected. Additionally, the peptide 364–374 on helix XI, which contains sugar-binding residues Gln372 and Thr373, was protected by Na^+^ alone (P = 0.029) or Na^+^ with melibiose (P = 0.011) and was weakly protected by melibiose but at a P value of 0.056.

In the presence of melibiose and Na^+^, the deprotections of overlapping peptides 36–47 (loop1–2) and 284–293 (loop8–9) with high confidence (P < 0.01) were obtained. Melibiose alone caused the deprotections of peptide 36–47 with medium confidence (P < 0.05) (Fig. 5b, red). Five deprotected regions induced solely by melibiose are found at the ends of transmembrane helices with extended loops (Fig. 5b, pink). Overlapping peptide 292–298 (Fig. 5, Cyan) showed opposite effects when comparing melibiose alone to melibiose with Na^+^.

### Allosteric effects of Na^+^ binding on sugar-binding residues.

**Fig. 6.**
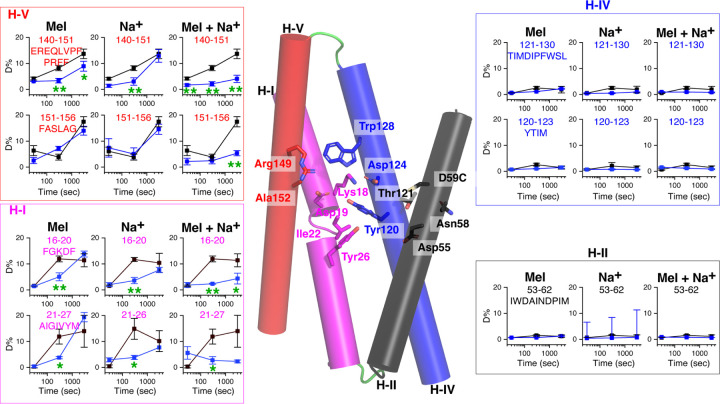
Allosteric effects on deuterium uptake by remote binding of Na^+^. The time course of percentage deuterium uptake was plotted against the labeling time at 0, 30, 300, and 3000 sec for the representative peptides that cover the residues responsible for the specificity of sugar or cation binding in MelB_St_. The peptide sequences are shown for each panel. *, statistically significant; **, statistically highly significant. Helices I and V (sugar-binding only), II (cation binding only), and IV (both), were shown in tube representation. Residues involved in sugar binding are represented by sticks and highlighted in magenta (helix I), blue (helix IV), and red (helix V). Residues involved in cation binding are highlighted in black. Labels in the time course and on the model are color-coded, except for the stick representing Thr121, which is black. Helices are labeled with Roman numerals and positioned at the beginning of each helix.

The helices II and IV, which host the coupling cations, were shown to have significantly lower deuterium uptake (Fig. 5b; Fig. S5) compared to helices I and V, which are specific for sugar binding and do not participate in cation binding. The effects of deuterium uptake by melibiose, Na^+^, or melibiose plus Na^+^ on the residues in the sugar- or Na^+^-binding pockets are summarized in Table S5. Seven peptides covering all N-terminal residues for sugar and cation binding were presented with the time courses of deuterium uptake. Notably, peptides 53–62, 120–123, and 121–130, which contain all cation-binding positions (Asp55, Asn58, Asp59, and Thr121), exhibited poor deuterium uptake rates, and their uptake rates were not significantly affected by the binding of melibiose, Na^+^, or both (Fig. 6, labeled in black (II) and blue (IV), respectively).

On the contrary, the two peptides (16–20 and 21–27) covering the sugar-binding residues 18, 19, 22, and 26 on helix I exhibited strong protection by melibiose, both in the absence and presence of Na^+^ (Fig. 6, magenta label). Remarkably, both peptides on helix I are also protected by Na^+^, which binds at helices II and IV, remotely. Additionally, the sugar-binding residue Arg149 on helix V was covered by several overlapping peptides that consistently demonstrated strong protections by melibiose, Na^+^, or melibiose plus Na^+^, as illustrated by peptide 140–151 (Fig. 6, red label). The residue 152, located in the binding pocket but not in direct contact with sugar, showed no detectable protection by melibiose or Na^+^ alone; however, significant protection was observed in the presence of both melibiose and Na^+^. The protective effects on the C-terminal sugar-binding residues Gln372 and Thr373 have been described; the peptide 369–374 is protected by Na^+^ alone and stronger protection was observed in the presence of melibiose plus Na^+^ (Fig. 5b).

## Discussion

The dehydration of sugar molecules is required for sugar binding, yet little is understood about this process in MelB. In the 2.7 Å-resolution α-NPG-bound structure, a water molecule was modeled to a positive density, and surrounded by the OH-4 and OH-6 groups on the galactopyranosyl ring, as well as by Gln372 and Thr373 on helix XI. In the published MelB_St_ structures with α-NPG or DDMB bound (20, 22) and the current structures with native melibiose, α-MG, or raffinose bound, due to the limited resolutions, the water molecule was not modeled; however, the positive density at the same position always appeared in the mFo-DFc maps, which indicate that this OH-4 water molecule is part of the sugar binding coordination in MelB.

The structures complexed with galactosides containing different numbers of sugar units, ranging from one to three, clearly demonstrate that the specificity is directed toward the galactosyl moiety (Figs. 1–3). The sugar-protein polar interactions primarily occur at positions 2, 3, 4, and 6. Notably, the orientation of the hydroxyl group at the C4 position is the crucial feature for distinguishing between galactose and glucose. The OH-4 on α-NPG is within hydrogen-bonding distances from the carboxyl group of Asp124 and the indole group of Trp128, and it indirectly connects with Gln372 and Thr373 on helix XI through this water molecule bound to OH-4 (Fig. 2). These interactions define the specificity of galactosyl molecules, as the glucose or glucoside OH-4 points to the opposite side of the galactosyl plane; thus, it does not form the hydrogen-bonding network around OH-4 and therefore would not be selected by MelB_St_. Clearly, the water molecule bound to OH-4 contributes to the sugar recognition.

Another important piece of structural information is that the OH-4 group and the bound water are in close proximity to the Na^+^-binding residue Thr121 and the cation-binding important residue Lys377, indicating that the OH-4 water molecule appears on the pathway between the two specificity-determining pockets. The OH-3 and OH-2 groups on the opposite edge of the galactopyranosyl ring create multiple hydrogen-bonding interactions with the charged residues Asp19 and Arg149. These interactions likely help to stabilize recognition at OH-4 and enhance the binding affinity.

All available structures of MelB_St_ (19–21) and MD simulations on the coupled translocation of melibiose and Na^+^ (26) indicate that there is no direct interaction between the bound Na^+^ and the bound galactosides. Binding affinity determinations of the primary substrate and the coupling cations demonstrate their positive cooperativity (19, 20, 27). The MD simulations suggested that Na^+^ binding allosterically affects the local electrostatic interactions in the sugar binding pocket. HDX-MS was used to obtain experimental evidence for the structural dynamics induced by melibiose, Na^+^, or melibiose combined with Na^+^.

HDX determines the structural motion or dynamics by measuring exchange rate over time(28). The resolution of this method is limited to the peptide level, and the deuterium uptake rate is affected by the interplay of structural dynamics and solvent accessibility. Previously, we used this technique to study the conformational dynamics between inward- and outward-facing states of MelB_St_ with bound Na^+^. In this study, we tested the Apo state and Holo states with different ligands. At Apo state, the peptides with faster deuterium uptake rates are distributed in both N- and C-terminal domains, and helices I and V are the three helices with the fastest uptake rates, indicating those regions at Apo state are more dynamic. The previous thermal denaturation study detected by circular dichroism spectroscopy showed that the stability of the Apo state is lower than that of any holo states, either with melibiose or Na^+^; the most stable state is the ternary form with both melibiose and Na^+^ bound(27). This HDX study identified the dynamic regions that contribute to the poor stability of Apo MelB_St_.

There is a significant difference in the dynamics of the sugar-binding helices (I, IV, V, X, and XI) and the cation-binding helices (II and IV). The cation-binding helix II (containing Asp55, Asn58 and Asp59) exhibits poor deuterium uptake and helix IV (containing Thr121) contains more non-covered positions either not covered by labelling or not covered by peptides. In either condition, their solvent accessibility and H/D exchange should be expected to be low. Therefore, the areas hosting the cation binding are relatively rigid (Figs. 5–6). In contrary, most sugar-binding helices, e.g., helices I (containing Lys18, Asp19, Ile22 and Try126), V (Arg149 and Ala152) and XI (containing Gln372 and Thr373), are dynamics; however, helix IV (containing Tyr120, Asp124, and Try128) that also carries the Na^+^-binding residue Thr121 is rigid. Notably, Asp19 and Arg149, which contribute to binding affinity, are located in the most dynamic helices I and V. Stabilization of both helices is expected to increase the affinity for sugar.

Melibiose-induced structural changes include dynamic restraints and enhancements (Fig. S5a). The structural restraints or protections were observed on the binding residue-hosting helices (I and V) and two loop helices (the middle loop and the C-terminal tail), while the deprotections affect a wide range of areas, which are mapped on the helices-loop regions and most are at the periplasmic side. Interestingly, most regions protected by melibiose are also protected by Na^+^ alone; in addition, the water-mediated sugar-binding residues Gln372 and Thr373 are also protected (Fig. S5b). There is no significant deprotection detected by Na^+^, consisting with the conclusion that the Na^+^-bound MelB is more stable than the Apo state.

When both melibiose and Na^+^ are presented during the H/D exchange reaction, most of the protected regions by melibiose or Na^+^ are also protected (Fig. S5c). Two deprotected regions were identified. One is also deprotected by melibiose, which is the peptide 36–47 located in Loop_1–2_ and the beginning of the helix II; another deprotected region 292–298 is on the extended helix VIII. Both regions become more dynamic in the ternary state with both melibiose and Na^+^. Notably, the deprotected region 292–298 contains Arg295, and two water molecules are modeled in the 2.70 Å map which stabilize the salt-bridge Arg295 and Asp351 on helix X, which is part of the large comprehensive charged network to stabilize the packing between the N- and C-halves at the cytoplasmic leaflet region for the inward-facing state. The binding of both prepared the transporter to initiate the conformational transition from the outward to the inward-facing states. Arg295 might respond to this binding signal and become more dynamics to destabilize the charged network and facilitate the opening at the cytoplasmic surface.

The MD simulation and free energy calculations and distance distribution indicated that the Asp19 is highly dynamic when Na^+^ unbound, and the binding of Na^+^ shifted the weak interaction of melibiose to stronger single interaction(26). HDX-MS data showed that the binding of Na^+^ alone causes the protections of the dynamic regions that carry Asp19 and Arg149, so the sugar-binding residues near OH-3 and OH-2 groups are more rigid. Therefore, it can be concluded that Na^+^ binding rigidifies the sugar-binding pocket through an allosteric effect on the local electrostatic interactions as proposed from the MD simulations.

The symporter H^+^-coupled lactose permease LacY and MelB share several common features (5, 31, 32). Both transporters share a similar substrate spectrum, and all are selective for galactosides. In addition, the cation-binding affinity in both LacY and MelB does not appear to be influenced by conformational transition(21, 32, 33). Furthermore, their cation sites do not have direct contact with their primary substrate galactosides. Glu325 of LacY is the sole protonation residue (34) and located in a hydrophobic milieu with no direct contact with galactosides(31, 33, 35). It has also been demonstrated that the sugar-binding affinity depends on the protonation status of LacY (36), which could be postulated to be an allosteric effect. The role of the coupling cation as an allosteric activator in cation-coupled symporters might be a common mechanism. Understanding this could provide broader insights into how these transport systems operate.

## Experimental Procedures

### Reagents.

Melibiose was purchased from *Acros Organics* (*ThermoFisher Scientific*). Raffinose (RPI Chemicals), α-methyl galactoside andα-nitrophenyl galactoside are purchased from Sigma-Aldrich. Detergents undecyl-β-D-maltopyranoside (UDM) and DDM (dodecyl-β-D-maltopyranoside (DDM) were purchased from *Anatrace*. *E. coli* lipids (Extract Polar, 100600) were purchased from *Avanti Polar Lipids, Inc*. All other materials were reagent grade and obtained from commercial sources.

### Strains and plasmids.

*E. coli* DW2 cells (*mel*A^+^, *melB*^*−*^*, lacZ*^*−*^*Y*^*−*^) (37) were used for protein expression and functional studies. The expression plasmids pK95/ΔAH/WT MelB_St_/CHis_10_(15) and pK95/ΔAH/D59CMelB_St_/CHis_10_ (18) were used for constitutive expression.

### MelB_St_ protein expression and purification.

Cell growth for the large-scale production of WT MelB_St_ or D59C MelB_St_ were carried out in *E. coli* DW2 cells (18, 38). Briefly, MelB_St_ purification by cobalt-affinity chromatography (Talon Superflow Metal Affinity Resin, Takara) after extraction by 1.5% UDM. MelB_St_ protein was eluted with 250 mM imidazole in a buffer containing 50 mM NaPi, pH 7.5, 200 mM NaCl, 0.035% UDM, and 10% glycerol, and further dialyzed to change the buffer conditions accordingly.

### Protein concentration assay.

The Micro BCA Protein Assay (Pierce Biotechnology, Inc.) was used to determine the protein concentration.

### Isothermal titration calorimetry.

All ITC ligand-binding assays were performed with the TA Instruments (Nano-ITC device) as described (19), which yields the exothermic binding as a positive peak. The MelB_St_ was dialyzed overnight with assay buffer containing 20 mM Tris-HCl (pH7.5), 100 mM NaCl, 10% glycerol and 0.035% UDM. The ligands are prepared by dissolving in the same batch of dialysis buffer for buffer matching. In a typical experiment, the titrand (MelB_St_) placed in the ITC Sample Cell was titrated with the specified titrant raffinose, α-methyl galactoside, melibiose, or α-NPG (placed in the Syringe) in the assay buffer by an incremental injection of 2.5-μL aliquots at an interval of 2500 sec at a constant stirring rate of 250 rpm (nano-ITC). MelB_St_ protein samples were buffer-matched to the assay buffer by dialysis. The normalized heat changes were subtracted from the heat of dilution elicited by the last few injections, where no further binding occurred, and the corrected heat changes were plotted against the mole ratio of titrant versus titrand. The values for the binding association constant (*K*_a_) were obtained by fitting the data using the one-site independent-binding model included in the NanoAnalyze software (version 3.7.5). The dissociation constant (*K*_d_) = 1/*K*_a_.

### Crystallization, native diffraction data collection, and processing.

The D59C MelB_St_ dialyzed overnight against the sugar-free dialysis buffer (20 mM Tris-HCl, pH 7.5, 100 mM NaCl, 0.035% UDM, and 10% glycerol), concentrated with Vivaspin column at 50 kDa cutoff, and stored at −80 °C. A phospholipid stock solution of 20 mM was prepared by dissolving the *E. coli* Extract Polar (Avanti, 100600) with a dialysis buffer containing 0.01% DDM. The protein sample was diluted to a final concentration of 10 mg/ml with the same sugar-free dialysis buffer, supplemented with phospholipids at 3.6 mM and 30 mM of melibiose or α-MG, 40 mM raffinose, or 6 mM of α-NPG in DMSO solution. Crystallization trials were carried out by the hanging-drop vapor-diffusion method at 23 °C by mixing 2-μL protein with 2-μL reservoir. Crystals from D59C MelBSt protein with the melibiose, α-MG, or α-NPG appeared against a reservoir consisting of 100 mM Tris-HCl, pH 8.5, 100 mM NaCl_2_, 50 mM CaCl_2_, and 32–35% PEG 400. For the raffinose-containing sample, the crystals were collected from 100 mM Tris-HCl, pH 8.5, 50 mM CaCl_2_, 50 mM BaCl_2_, and 32.5% PEG 400. All crystals were frozen with liquid nitrogen in 2 weeks, and tested for X-ray diffraction at the Lawrence Berkeley National Laboratory ALS BL 5.0.1 (dataset for melibiose, α-MG, and α-NPG-containing complex) or 5.0.2 (raffinose-containing datasets) via remote data collection method.

ALS auto-processing XDS or DIALS programs output files were further reduction by AIMLESS in the ccp4i2 program for the structure solution (39). The statistics in data collection are described in Table S1.

### Structure determination.

The structure determination was performed by the Molecular Replacement method using the α-NPG-bound D59C MelB_St_ mutant structure [PDB ID 7L17] as the search template, followed by rounds of manual building and refinement to resolutions of 2.70 Å, 3.05, 3.45, or 3.68 Å for structure with α-NPG, melibiose, raffinose, or α-MG, respectively, in Phenix (40). The structures were modeled from positions 2 to 453 or 456, respectively, without a gap, and the missing side chains due to the density disorder, and Ramachandran assessment were listed in the Tables S6.

### Sugar docking and modeling.

A strong positive density, with varying sizes and shapes, was observed in the difference maps of each of the four structures. The size and shape matched the sugars that co-crystallized, and the docked sugar molecules fitted well with the densities. The sugar refinement restrains were generated from SMILES using the ELBOW program in Phenix. To the 2.7 Å α-NPG-bound map, six water molecules were modeled. In addition, a PEG molecule (ligand ID 1PE) was also model to a strong positive density with a sausage shape aligning with the helix IX.

### Hydrogen-deuterium exchange coupled to mass spectrometry (HDX-MS).

In-solution HDX-MS experiment was performed to study the substrate-induced structural dynamics of MelB_St_. As described (21), the labeling, quenching, lipids removal, and online digestion were achieved using a fully automated manner using a HDx3 extended parallel system (LEAP Technologies, Morrisville, NC)(41, 42). MelB_St_ was prepared at 50.0 μM in a Na^+^-free buffer (25 mM Tris-HCl, pH 7.5, 150 mM choline chloride, 10% glycerol, 0.035% UDM in H_2_O), either in the absence of a substrate (Apo) or in the presence of 100 mM melibiose, 100 mM NaCl, or both. The hydrogen/deuterium exchange reaction as described previously (21). Briefly, aliquots of 4 μl of each sample were diluted10-fold into the labeling buffer (25 mM Tris-HCl, pD 7.5, 50 mM choline chloride, 10% glycerol, 0.035% UDM in D_2_O), without or with 100 mM of melibiose, Na^+^, or both. Labeled samples were incubated in D_2_O buffer at 20 °C for multiple time points (30 sec, 300 sec, and 3000 sec) in triplicates and non-deuterated controls were prepared in a similar manner except H_2_O buffer was used in the labeling step.

At each designated time point, reaction was quenched by adding an equal volume of ice-cold quench buffer (6 M urea, 100 mM citric acid, pH, 2.3 in H_2_O) for 180 seconds at 0 °C and immediately subjected to a lipid filtration module integrated on the LEAP PAL system. After incubation of a 60 sec with ZrO2 particles, the LEAP X-Press then compressed the filter assembly to separate proteins from the ZrO2 particles-bound phospholipids and detergents. The filtered protein sample was injected into a cool box for online digestion and separation.

LC/MS bottom-up HDX was performed using a Thermo Scientific^™^ Ultimate^™^ 3000 UHPLC system and Thermo Scientific^™^ Orbitrap Eclipse^™^ Tribrid^™^ mass spectrometer. Samples were digested with a Nepenthesin-2 (Affipro, Czech Republic) column at 8 °C and then trapped in a 1.0 mm x 5.0 mm, 5.0 μm trap cartridge for desalting over 180 sec. The resulting peptides were then separated on a Thermo Scientific^™^ Hypersil Gold^™^, 50 ×1 mm, 1.9 μm, C18 column with a gradient of 10 % to 40 % gradient (A: water, 0.1 % formic acid; B: acetonitrile, 0.1 % formic acid) for 15 minutes at a flow rate of 40 μL/min. A pepsin wash was added in between runs to minimize the carryover.

A nonspecific digested peptide database has been created for MelB_St_ with a separate MS/MS measurement of non-deuterated samples as described (21). Digested peptides from undeuterated MelB_St_ protein were identified on the orbitrap mass spectrometer using the same LC gradient as the HDX-MS experiment. Using the Thermo BioPharma Finder software (v 5.1), MS2 spectra were matched to the MelB_St_ sequence with fixed modifications.

A number of 146 or 150 peptide assignments (confident HDX data cross all labeling times) were confirmed for MelB_St_ samples giving 86–87% sequence coverage. The MS data were processed using the Sierra Analytics HDExaminer software with the MelB_St_ peptide database. Following the automated HDX-MS analysis, manual curation was performed. Upon the completion of the data review, a single charge state with high-quality spectra for all replicates across all HDX labeling times was chosen to represent HDX for each peptide. Differential HDX data were tested for statistical significance using the hybrid significance testing criteria method with an in-house MATLAB script, where the HDX differences at different protein states were calculated (ΔD = D_Holo_ - D_Apo_). Mean HDX differences from the three replicates were assigned as significant according to the hybrid criteria based on the pooled standard deviation and Welch’s t-test with P < 0.05. The statistically significant differences observed at each residue (D_Mel - Apo_ < |0.186|, ΔD_(Na+)_ - Apo < |0.224|, or ΔD_Na(+)Mel_ - Apo < |0.175|) were used to map HDX consensus effects based on overlapping peptides onto the structure models.

### Statistics and reproducibility.

All experiments were performed 2–4 times. The average values were presented in the table with standard errors. An unpaired t-test was used for statistical analysis.

### Graphs.

Pymol (3.1.5.1) (43) and UCSF ChimeraX (X) were used to generate all graphs. The program Origin 2024 was used to plot the ITC curves.

## Data Availability

The x-ray diffraction datasets and models have been deposited to wwPDB under the accession codes 9OLD for the α-nitrophenyl galactoside-bound, 9OLI for the melibiose-bound, 9OLR for the α-methyl galactoside, as well as 9OLP for the raffinose-bound D59C MelB_St_.

## Abbreviations:

MelB_St_: *Salmonella enterica* serovar Typhimurium melibiose permease
MFS: major facilitator superfamily
ITC: isothermal titration calorimetry
α-NPG: 4-nitrophenyl-α-D-galactopyranoside
Mel: melibiose
α-MG: α-methyl galactoside
Raff: raffinose
*K*_d_: dissociation constant
HDX-MS: hydrogen-deuterium exchange coupled to mass spectrometry
D: deuterium
